# High-Reflective Templated Cholesteric Liquid Crystal Filters

**DOI:** 10.3390/molecules26226889

**Published:** 2021-11-15

**Authors:** Yao Gao, Yuxiang Luo, Jiangang Lu

**Affiliations:** National Engineering Lab for TFT-LCD Materials and Technologies, Department of Electronic, Engineering, Shanghai Jiao Tong University, Shanghai 200240, China; gaoyao123@sjtu.edu.cn (Y.G.); sjtu_lyx@sjtu.edu.cn (Y.L.)

**Keywords:** cholesteric liquid crystals, high reflectivity, single layer, templating technology

## Abstract

Cholesteric liquid crystals (CLCs) have been widely applied in optical filters due to Bragg reflection caused by their helical structure. However, the reflectivity of CLC filters is relatively low, commonly less than 50%, as the filters can only reflect light polarized circularly either left- or right-handedly. Therefore, a high-reflective CLC filter with a single-layer template was proposed which may reflect both right- and left-handed polarized light. The CLC filters of the red, green, blue color were fabricated by the templating technology, which show good wavelength consistency. Additionally, a multi-phase liquid crystal filter with high reflectance was demonstrated by the single-layer templating technology. The templated CLC or multi-phase liquid crystal filters show great potential applications in the optical community, reflective display, and lasing.

## 1. Introduction

Optical filters with liquid crystal (LC) materials have attracted significant attention in the optical community and color display [1,2]. Various types of optical filters, such as Bragg grating filters, color filters, and waveguide filters, have been developed [3,4,5,6]. For the LC materials among optical filters, cholesteric liquid crystals (CLCs) have been widely applied due to their self-assembled structures and simple fabrication [7]. The CLCs possess the intrinsic features of pitch- and polarization-selective reflection of the incident light [8,9,10,11,12]. For pitch-selective reflection of CLCs, the reflection central wavelength is determined by p and n, where p is the pitch length which is determined by chiral dopant concentration and helical twist power (HTP), n = (n0 + ne)/2, n0 and ne are the ordinary and extraordinary refractive indices of LC, respectively [13,14,15,16,17]. Different pitch length, leading to the different reflection central wavelength that covers from ultraviolet to infrared, can be achieved by changing the chiral dopant concentration. Because the reflection by the CLCs with a helical structure is selective, only circularly polarized incident light with the same handedness as that of CLC is reflected, while light with the opposite handedness is transmitted. Accordingly, for the unpolarized or linearly polarized incident light, the reflectance of a single CLC film is equal to, at most, 50% [11,18]. However, a higher reflectivity of a CLC device is desired to apply in many fields, including reflective display, lasing and mirrors, especially in optical filters.

To improve the reflectance of CLC devices, several approaches have been proposed. A single layer of CLC materials whose optical characteristic go beyond the 50% reflectance limit was first proposed. The CLC exhibited a thermally induced helicity inversion by introducing photopolymerizable monomers and the reflectance exceeded 50% when measured at the temperature assigned at a cholesteric helix with the same pitch but a left-handed sense before reaction [12,19]. A stack of two individual single-chiral CLC cells which can reflect both right-and left-circularly polarized light was proposed [20,21]. One wash out–refilling process that functions by refilling optical adhesive into a cholesteric film, assembled utilizing two cholesteric templates with opposite handedness, was proposed [22]. In addition, a half-wave plate, converting the single-handedness polarized light to opposite handedness light, could be used to insert between two separated CLCs with the same handedness [23,24]. Although effective in realizing high-reflective CLC devices, the approaches mentioned above suffer from complicated design and fabrication, increased insertion loss and optical defects at the interfaces, and increased weight [10,25]. In this paper, we demonstrate CLC filters with high-reflectivity based on a single-layer helical structure and simple fabrication process. The filters are formed via CLC templating technology with a wash out–refill process which functions by refilling the CLC into a single layer CLC template with opposite handedness [10,26,27]. This templating technology is effective in realizing high-reflectivity single-layer CLC filters for all red, green, and blue colors. Compared with the approaches mentioned above, the preparation process of fabricated CLC filters is simple and applicable to large-scale application. According to our previous study where the effect of multi-phase liquid crystals was investigated [28], the multi-phase liquid crystal filters with high reflectivity are demonstrated in this paper. By refilling a CLC with left-handedness into a blue phase liquid crystal (BPLC) template with right-handedness, a multi-chiral BPLC filter with a single layer was fabricated. Herein, the fabrication of the high-reflectivity CLC or BPLC filters with a single layer show great potential for applications such as flexible reflective displays, optical communication, lasing, and color filters.

## 2. Experimental Design and Sample Fabrication

### 2.1. Experimental Design

In our previous work, a multi-phase or multi-pitch twist structure LC can be fabricated by the wash out–refilling process [28]. Therefore, in this study, we have considered that the multi-chiral CLC filters with high reflectivity can be obtained by the wash out–refilling process. For achieving the purpose, the polymer-stabilized CLC(PS-CLC) precursors with right-handedness, using the different concentration chiral dopant to reflect light of different colors, were used to make the cholesteric templates with single layer and the left-handed CLCs used as refilling materials were prepared. The polymer networks with right-handedness were formed by wash-out process and, after refilling the left-handed CLCs into the templates, the two handedness could co-exist in a device which may reflect both right- and left-circularly polarized light.

**Figure 1:**
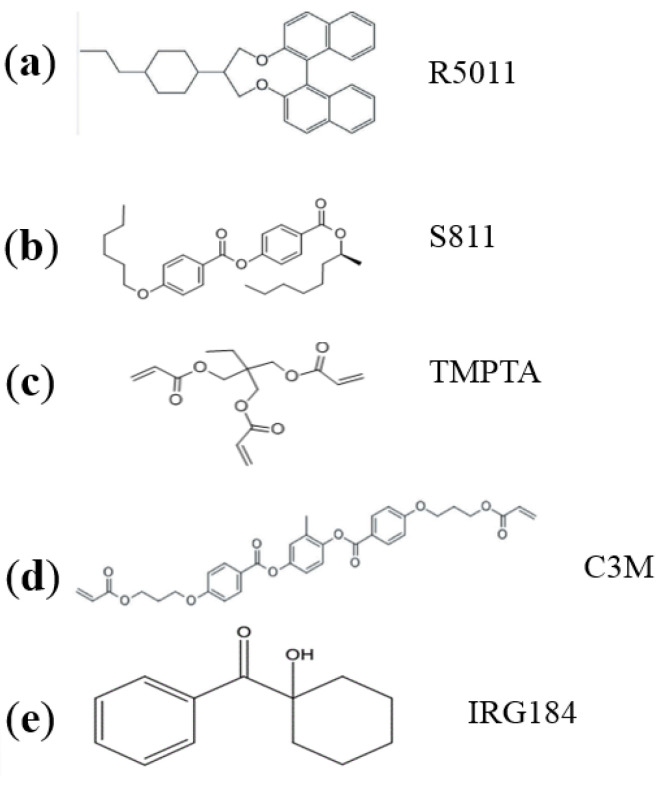
Materials used in our experiments and their chemical structures: (**a**) R5011, (**b**) S811, (**c**) TMPTA, (**d**) C3M, (**e**) IRG184.

The materials prepared for our experiments include the following components: a positive nematic liquid crystal (BPH006, Jiangsu Hecheng Display Technology Co., Ltd. (HCCH), Nanjing, Jiangsu, China), a chiral dopant with right-handedness (R5011, Nanjing Murun, Nanjing, Jiangsu, China), a chiral dopant with left-handedness (S811, HCCH), an ultraviolet (UV) curable monomer (TMPTA, Shanghai Macklin, Shanghai, China), a cross-linker agent (C3M, HCCH), and a photo-initiator (IRG184, HCCH). The chemical structures of these materials are shown in Figure 1. The mixtures of PS-CLC precursors with right-handedness were composed of BPH006, R5011, TMPTA, C3M, and IRG184, and the CLC mixtures with left-handedness were composed of BPH006 and S811. Because the value of HTP of S811 is very small compared to R5011, the concentration of S811 needs to be high enough to obtain the left-handed CLC whose reflective peak is the same as the templated-CLC (T-CLC) with right-handedness. Table 1 lists PS-CLC materials with different right-handed chiral dopant concentration and CLC materials with different left-handed chiral dopant concentration composition corresponding to red, green, and blue colors.

### 2.2. Sample Fabrication

**Figure 2:**
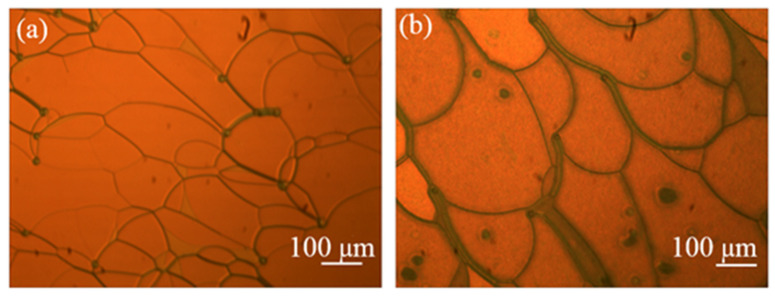
The surface morphology of (**a**) PS-CLC and (**b**) T-CLC of red color.

**Figure 3:**
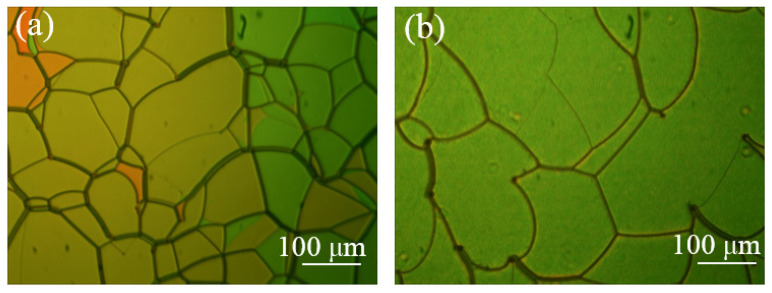
The surface morphology of (**a**) PS-CLC and (**b**) T-CLC of green color.

**Figure 4:**
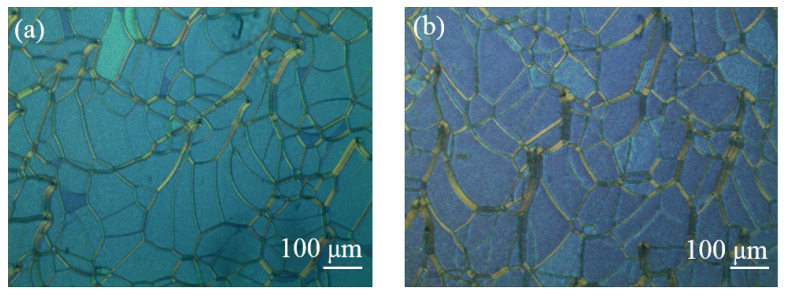
The surface morphology of (**a**) PS-CLC and (**b**) T-CLC of blue color.

In order to obtain the multi-chiral CLCs reflecting both right- and left-circularly polarized light, first the materials in Table 1 were added into the containers and were fully stirred on the thermostatic magnetic stirrer (524G, Shanghai Messrs Instrument, Shanghai, China) for 10 min, respectively, until the phenomenon of clear and transparent liquid appeared which means the mixture of the PS-CLC precursors and left-handed CLCs we desired were obtained. Then, the PS-CLC precursors and left-handed CLCs were capillary infused into empty cells of 8 μm thickness and 25 mm × 20 mm area with antiparallel alignment at 80 °C on the temperature controller (HCS302, Instec Co., Boulder, CO, USA), respectively. Then, the PS-CLC precursors and CLCs were cooled down to 30 °C, which was determined by the LC phase state at a rate of 0.5 °C/min by utilizing the temperature controller, and the phase transition process and platelet texture of these mixtures were observed under a polarized optical microscope (POM, XPL-30TF, Shanghai WeiTu Optics & Electron Technology Co., Ltd., Shanghai, China). Finally, the PS-CLCs were obtained by ultraviolet exposure with a wavelength of 365 nm and intensity of 3 mw/cm^2^ for 10 min. The platelet texture of PS-CLC of the red, green, and blue color are shown in Figure 2, Figure 3 and Figure 4a.

**Figure 5:**
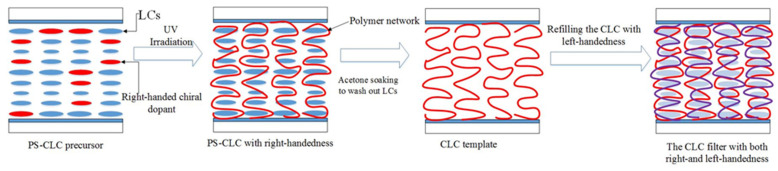
The fabrication process of multi-chiral CLC.

To obtain the templated-CLC(T-CLC), we put the PS-CLC cells of red, green, and blue colors in acetone for about 48 h to wash out the unpolymerized components, including LC, chiral dopant, unreacted monomers, and photo initiator. The washing rate was not high due to the upper and lower glass substrates of the cells not being separated, causing the washing time to be long enough. After that, the cells with CLC template were placed on the temperature controller with 80 °C to bake the residual acetone. Then a nematic liquid crystal, BPH006, was refilled into the CLC template at 80 °C and cooled down to 30 °C at a rate of 0.5 °C/min, the T-CLCs were obtained and the surface morphology of T-CLC could be observed, as shown in Figure 2, Figure 3 and Figure 4b. Finally, the three kinds of CLCs with different central wavelength with a close peak distance to the T-CLC of red, green, and blue color were refilled into the three CLC templates at 80 °C on the temperature controller, respectively. Subsequently, the temperature was decreased to 30 °C at a rate of 0.5 °C/min and the multi-chiral CLC filters of red, green, and blue color were fabricated. The fabrication process of multi-chiral CLC filters is shown in Figure 5.

## 3. Measurement and Discussion

**Figure 6:**
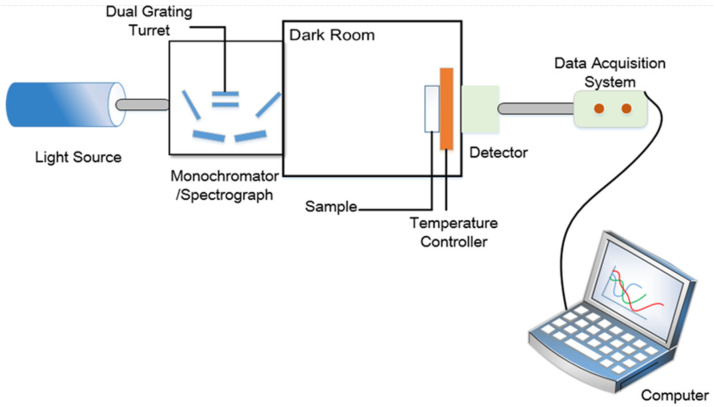
Schematic diagram of transmittance measurement device.

The spectral characteristics of CLC filters can be analyzed by equipping the measurement system which is shown in Figure 6. All the Bragg reflection spectra were measured by unpolarized light provided by a tungsten bromide lamp with the whole visible light range. The unpolarized light passing through a monochromator (Omni-λ1509, Zolix, Beijing, China) was incident on the sample, and the intensity signals of incident light passing through the sample were collected by a data acquisition system (DCS300PA, Zolix, Beijing, China) utilizing an optical fiber. Since the devices were fixed on the position shown in Figure 6, we characterized the properties of templated cholesteric liquid crystal filters with transmittance spectra. The transmittance was calculated as the ratio of the measured light intensity in the measurement system with a sample cell to that with an empty cell. Although the transmittance of the transmittance spectra may be decreased by scattering or absorbing, the central wavelength and the maximum transmissivity/reflectivity of the transmittance and reflection spectra had almost the same values.

**Figure 7:**
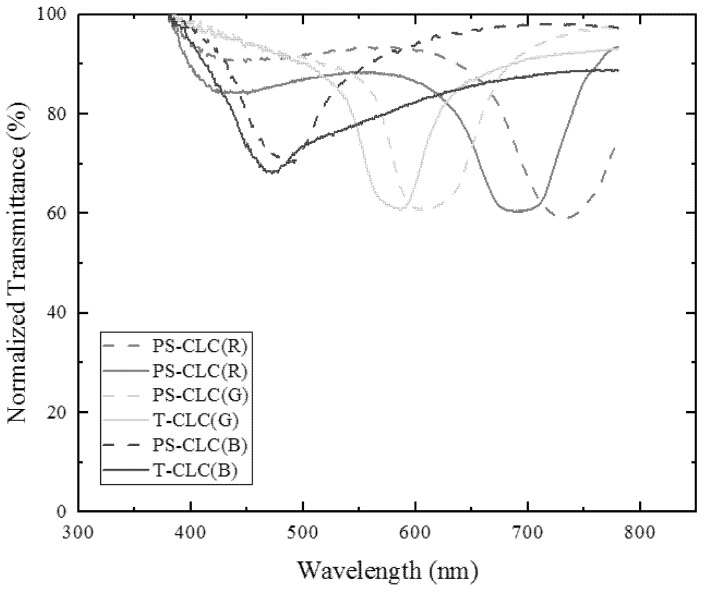
The normalized transmission spectrum of polymer-stabilized cholesteric liquid crystals (PS-CLCs) and templated cholesteric liquid crystals (T-CLCs) for red, green, and blue colors.

With the different chiral dopant concentrations shown in Table 1, the transmission spectra of PS-CLCs and T-CLCs for red, green, and blue colors were achieved by the measurement system shown in Figure 6. As illustrated in Figure 7, the reflection central wavelength of PS-CLCs for red, green, and blue light are 735 nm, 603 nm, and 486 nm, respectively. After the wash-out and refilling process, the reflection central wavelength of T-CLCs were 689 nm for red, 587 nm for green, and 472 nm for blue colors, respectively. As we can see, the reflection central wavelength of T-CLCs appears a little blue shift after templating technology, as the pitch length of polymers has a slight decrease after the wash-out and refilling process. In addition, the result shows that the degree of decrease varies uniformly with color bands, the reflection central wavelength of T-CLCs has a larger blue shift for red light, compared to the blue light. The reason may be that the pitch length of the polymer network in the red color is larger, the volume shrinkage is more severe through the wash-out and refilling process, causing the degree of decrease to be larger. This result also shows that the CLC can be reconstructed well by the templating process.

**Figure 8:**
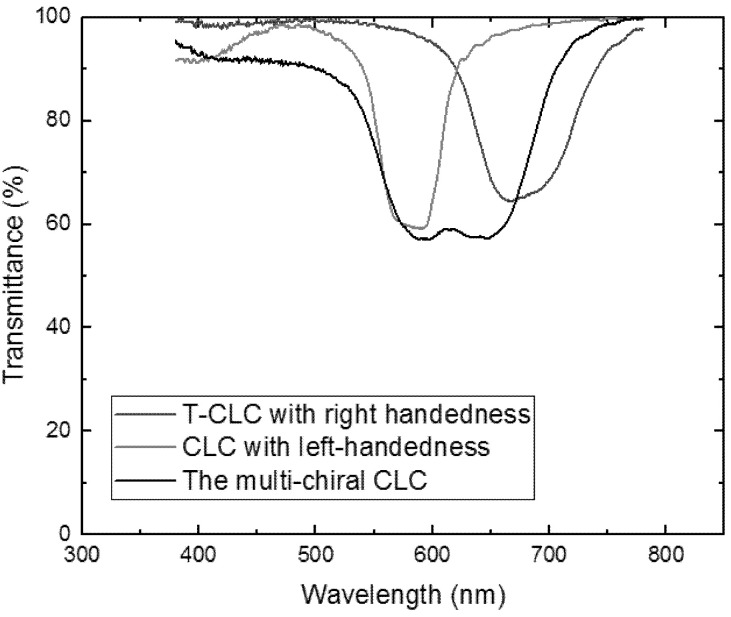
Transmission spectra of multi-chiral CLC with different central wavelength of T-CLC and CLC.

If the reflection central wavelengths of T-CLCs with right-handedness and CLCs with left-handedness were not superposed, the reflectivity of fabricated multi-chiral CLC filters with a single layer by templating technology would not have been greatly improved, as shown in Figure 8. We can observe that the reflectivity of the multi-chiral CLC filter was less than 50% after the wash out–refilling process, with two different central wavelengths and single chiral CLCs. The result also demonstrates that if the right-handed CLC template was refilled with a left-handed CLC of different pitch, the two reflection peaks were obtained. Therefore, the reflection wavelength of T-CLC must be same as the CLC to achieve the high-reflective CLC filters with multi-chiral.

**Figure 9:**
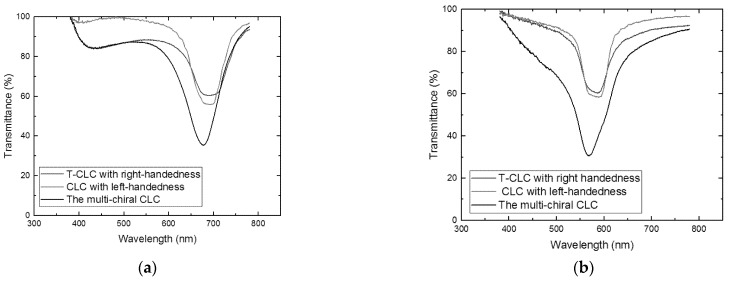

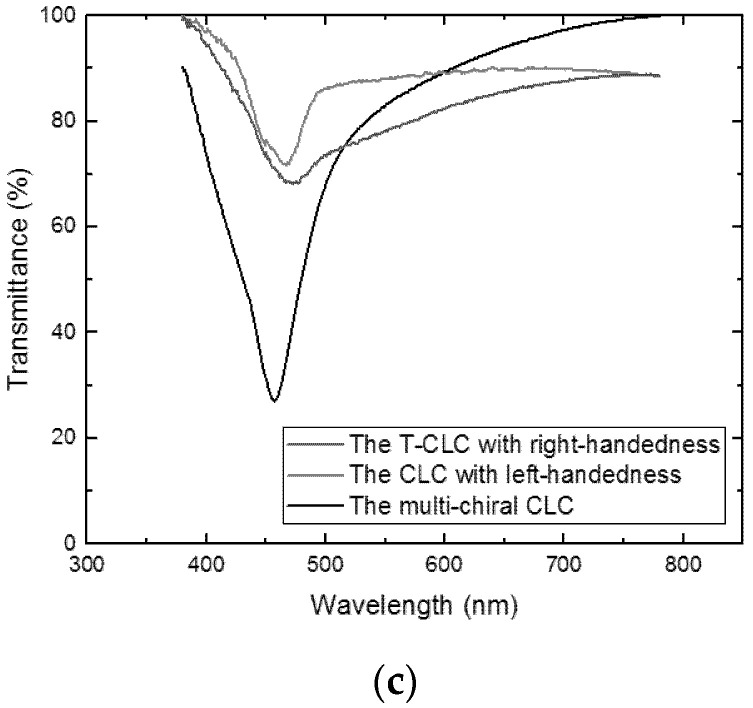
Transmission spectra of multi-chiral CLC filters with single layer, corresponding to the reflective (**a**) red, (**b**) green, and (**c**) blue color, respectively.

As shown in Figure 9a–c, it depicts the high-reflective CLC filters with the single layer fabricated refilling the right-handed T-CLC with left-handed CLC. The transmission spectra of high-reflective CLC filters corresponding to either a red, green, or blue reflective color were demonstrated, respectively. In order to obtain multi-chiral CLC filters with high-reflectivity for different colors, the concentration of the chiral dopants with right-handedness and left-handedness were adjusted to ensure that the central wavelengths of transmittance spectra of the T-CLCs with right-handedness and CLCs with left-handedness were approximately superposed. The blue and red curves represent the transmission spectra of the right-handed and left-handed CLC with a single layer, respectively. The transmission spectrum of T-CLC(R), T-CLC(G), T-CLC(B), (CLC(R), CLC(G), CLC(B)) is centered at 689 nm, 587 nm, and 472 nm (690 nm, 588 nm, and 467 nm), respectively. After the wash-out and refilling process, the transmission spectrum of the multi-chiral CLC filter was measured and is represented by the black curve in Figure 9a–c. As we can see, the reflection central wavelength of the multi-chiral CLC filters is 678 nm for red, 568 nm for green, and 457 nm for blue colors, respectively. In addition, the maximal reflectance of the multi-chiral CLC filters is 65% for red, 69% for green, and 72% for blue colors, which has improved by 63%, 75%, and 128%, compared to the reflectivity of T-CLCs with right handedness, respectively. The result also shows that the reflection central wavelength of multi-chiral CLC filters, compared to the single-chiral CLC (right-handed or left-handed CLC), appear to have a slight change of blue shift. The result is attributable to the slight decrease in helical pitch of chiral polymer following the volume shrinkage through the wash-out and refilling process. In addition, the maximal reflectance of the multi-chiral CLC filter is not as high as the theoretical value, owing to the central wavelength of right-handed and left-handed CLC not being fully superposed. To sum up, the smaller the offset of central wavelength between the right-handed and left-handed CLC filter, the larger the reflectivity of the multi-chiral CLC filter. Compared with the previous reported hyper-reflectivity film composed of at least two layers, the proposed single layer CLC filter with high reflectivity possesses distinguished advantages of simple fabrication, compact, and covering visible range.

**Figure 10:**
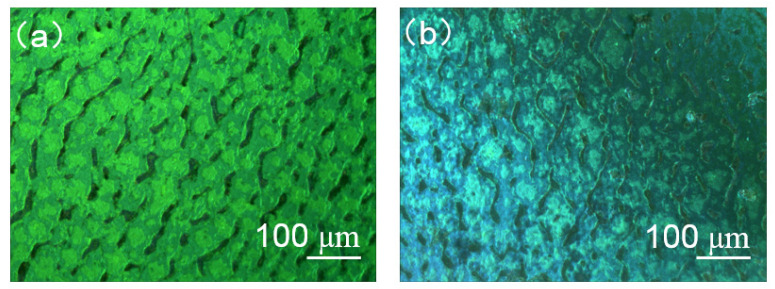
The surface morphology of (**a**) PS-BPLC and (**b**) T-BPLC.

**Figure 11:**
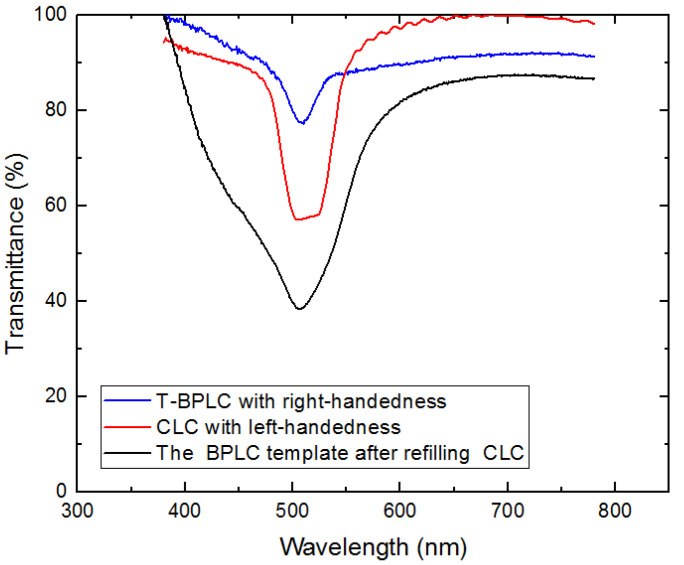
Transmission spectra of multi-chiral BPLC filters with single layer.

In our previous study, a multi-phase twist structure LC was fabricated. Therefore, the reflectivity improvement properties of multi-phase LC filters were investigated in this work. A multi-chiral BPLC filter was fabricated by utilizing templating technology used in the part of *Sample Fabrication*. It is noticed that the concentrations of materials used to fabricate polymer-stabilized blue phase liquid crystal (PS-BPLC) precursor with right-handedness and CLC with left-handedness were different from PS-CLC. The mixture of PS-BPLC precursor with right-handedness contains BPH006/R5011/TMPTA/C3M/IRG184 (weight ratio 84.8/3.39/4.87/6.84/0.10), and a left-handed CLC mixture contains BPH006/S811 (weight ratio 76.4/23.6). In addition, the polymerization temperature of PS-BPLC is 54 °C, which is different from 30 °C for PS-CLC. The platelet texture of PS-BPLC and templated-BPLC (T-BPLC) can be observed under a polarized optical microscope (POM, XPL-30TF, Shanghai WeiTu Optics & Electron Technology Co., Ltd., Shanghai, China), as shown in Figure 10a,b. The multi-chiral BPLC filter with single layer was fabricated by refilling the left-handed CLC into the right-handed T-BPLC, and the corresponding transmission spectra was achieved by the measurement system shown in Figure 6. As we can see in Figure 11, the blue and red curves represent the transmission spectra of the right-handed T-BPLC and left-handed CLC with a single layer, respectively. The central wavelengths of right-handed T-BPLC and left-handed CLC were 510 nm and 505 nm, respectively. After the wash-out and refilling process, the transmission spectrum of the multi-phase BPLC filter reflecting both right- and left-circularly polarized light was measured, which is represented by the black curve. The transmission spectrum of the multi-chiral BPLC filter is centered at 506 nm, and the corresponding maximal reflectance has improved, compared to the maximal reflectance of T-BPLC with right-handedness or CLC with left-handedness. Accordingly, for the multi-phase LC filter with single layer, coexisting both right- and left- handedness, the reflectivity can be improved compared with the single-handed LC filter. We can suppose that the reflectivity of the multi-chiral sphere phase LC fabricated by refilling a CLC with left-handedness into a sphere phase template with right-handedness may have also improved. 

## 4. Conclusions

In conclusion, a novel high-reflectivity CLC filter with a single layer template was proposed, which may reflect both right- and left-circularly polarized light. The templated CLC filters with a single layer of red, green, and blue color were fabricated in the wash out–refilling process, and the improvement of reflectivity was confirmed to be connect to the wavelength consistence. Moreover, a high-reflectivity multi-phase BPLC filter with a single layer template was achieved, which means that the technology for enhancing reflectivity applies equally to multi-phase LC filters with a single layer. The single layer templated CLC or BPLC filters show a simple process and great potential applications in the optical community, display, and lasing.

## Figures and Tables

**Table 1:** The PS-CLC precursors and CLCs with different chiral dopant concentrations.

Color	Sample	BPH006 [wt%]	R5011 [wt%]	S811 [wt%]	TMPTA [wt%]	C3M [wt%]	IRG184 [wt%]
Red	PS-CLC	86	1.45		5.00	7.45	0.10
	CLC	81.4		16.8			
Green	PS-CLC	86.13	1.8		5.13	6.84	0.10
	CLC	79.6		20.4			
Blue	PS-CLC	85.63	2.2		5.43	6.64	0.10
	CLC	74.6		25.4			

## Data Availability

Not applicable.

## Sample Availability

Samples of the compounds are available from the authors.
